# Joint spatiotemporal modelling of tuberculosis and human immunodeficiency virus in Ethiopia using a Bayesian hierarchical approach

**DOI:** 10.1186/s12889-024-20996-7

**Published:** 2025-01-30

**Authors:** Legesse Kassa Debusho, Leta Lencha Gemechu

**Affiliations:** Department of Statistics, University of South Africa, c/o Christiaan de Wet Road & Pioneer Avenue, Private Bag X6, Florida, 1710 Johannesburg, South Africa

**Keywords:** Bayesian hierarchical, HIV, Poisson regression, Relative risk, Joint spatiotemporal modelling, Tuberculosis

## Abstract

**Background:**

The aim of this paper was to evaluate the distribution of HIV and TB in Ethiopia during four years (2015-2018) at the district level, considering both spatial and temporal patterns.

**Methods:**

Consolidated data on the count of TB case notifications and the number of patients with HIV for four years, 2015-2018, were provided by the Ethiopian Federal Ministry of Health. The data was analyzed using the Bayesian hierarchical approach, employing joint spatiotemporal modelling. The integrated nested Laplace approximation available in the R-INLA package was used to fit six models, each with different priors, for the precision parameters of the random effects variances. The best-fitting model with the best predictive capacity was selected using the Deviance Information Criterion and the negative sum of cross-validatory predictive log-likelihood.

**Results:**

According to the findings of the selected model, about 53% of the variability in TB and HIV incidences in the study period was explained by the shared temporal component, disease-specific spatial effect of HIV, and space-time interaction effect. The shared temporal trend and disease-specific temporal trend of HIV risk showed a slight upward trend between 2015 and 2017, followed by a slight decrease in 2018. However, the disease-specific temporal trend of TB risk had almost constant trend with minimal variation over the study period. The distribution of the shared relative risks was similar to the distribution of disease-specific TB relative risk, whereas that of HIV had more districts as high-risk areas.

**Conclusions:**

The study showed the spatial similarity in the distribution of HIV and TB case notifications in specific districts within various provinces. Moreover, the shared relative risks exhibit a temporal pattern and spatial distribution that closely resemble those of the relative risks specific to HIV illness. The existence of districts with shared relative risks implies the need for collaborative surveillance of HIV and TB, as well as integrated interventions to control the two diseases jointly.

**Supplementary Information:**

The online version contains supplementary material available at 10.1186/s12889-024-20996-7.

## Background

Human immunodeficiency virus (HIV) and tuberculosis (TB) are epidemiologically associated [1]. The observed co-dynamics indicate a direct relationship between the two diseases, both *at the population level* [2] and *within the host* [3]. Despite being a disease that may be prevented and treated, TB remains the primary cause of mortality for individuals living with HIV [4]. The HIV pandemic has significantly influenced the rates of TB incidence as well [5]. It is predicted that people living with HIV (PLHIV) have a twentyfold increased likelihood of getting active TB in comparison to those who do not have HIV [6].

According to a study conducted in Ethiopia, there is a significant relationship between the occurrence of TB and the level of HIV infection [7]. Following the WHO recommendation, the Ethiopian government has implemented various strategies, for example expansion of the integrated service for TB and HIV to the health care facilities, to manage TB and HIV [8]. Despite the implemented interventions, Ethiopia continues to be one of the 30 countries identified in the 2021 WHO Global TB Report as having a high prevalence of TB and HIV from 2015 to 2020 [9].

TB and HIV are generally related diseases; therefore, their geographical patterns should hypothetically show standard features. Association between these patterns may serve as a second source of dependence to improve risk estimates of these diseases [10]. The spatiotemporal analysis enables us to investigate the continuation of a disease pattern throughout time simultaneously and can also help in identifying atypical disease patterns. The continuation could point to some potential factors that could impact prevalence of the disease, such as environmental factors, which assist in the interpretation of spatial patterns. Within the same spatiotemporal analysis, it is also possible to include space-time interaction terms to investigate the presence of localized clusters that may link to some environmental factors, strengthening the statistical inferences [11].

When conducting joint disease mapping research, each spatial unit is associated with at least two outcomes, such as the incidence of two diseases instead of only one. Since joint models use other additional response variables as an additional information source, this could improve risk estimates. Suppose there is a dependence between the two diseases. In that case, their dependence leads to sharing information between the two outcomes in the modeling process, which could improve risk estimates [10]. Various researchers in the disease mapping field have focused on applying the Bayesian joint spatial and spatiotemporal modeling of multiple diseases, see, e.g., [11–17]. Multiple studies conducted in Ethiopia have examined, separately at various levels, the spatial clustering and temporal trend of HIV and TB [18–23]. However, study to assess the joint spatiotemporal pattern of HIV and TB incidences and to identify locations with high-risk somewhat lacking in the country. This paper aimed to assess the distribution of HIV and TB relative risk in Ethiopia during four years (2015–2018) at the district level, considering both spatial and temporal patterns. The modeling framework in the statistical analysis was formulated via the Bayesian hierarchical modeling approach. The Bayesian hierarchical joint spatiotemporal modeling (BHJSTM) of two related diseases helps to strengthen inference as the modeling allows the borrowing of information between diseases. The BHJSTM utilises the combined power of data from several districts and years to generate smoothed estimates at the district level for each year. It also enables the investigation of geographical and temporal heterogeneity. In addition, it helps quantify the anticipated heterogeneity linked to potential risk factors and extract distinct patterns associated with each disease under investigation from the residual variations [11].

## Methods

### Study area

**Fig. 1:**
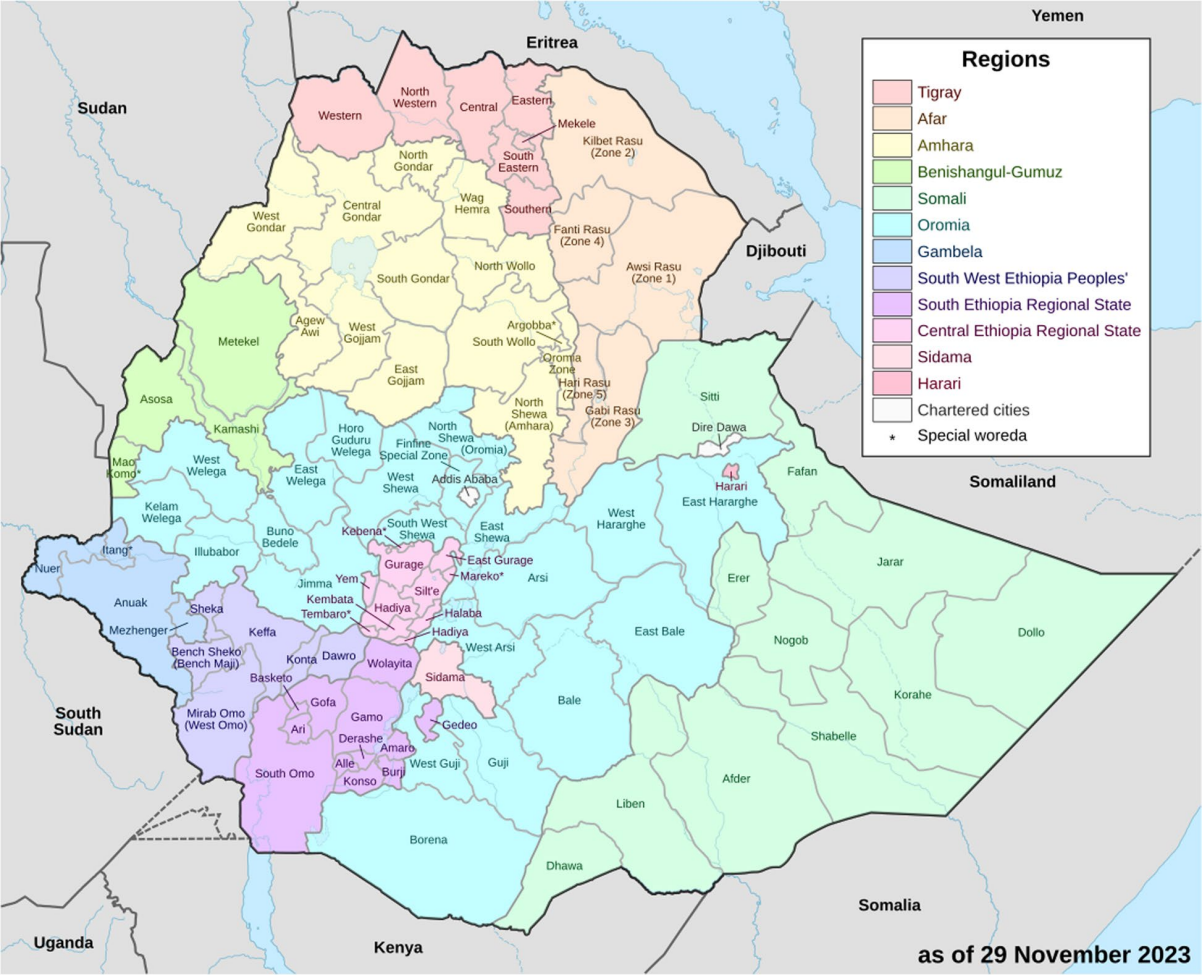
Geographical maps showing Ethiopia’s various zones and provinces. The abbreviations E, N, S, W, C, and Sp respectively represent east, north, south, west, northwest, and special

Ethiopia is situated in Africa’s northeastern region. The country’s administrative divisions, before 2021, were two city administrations, Addis Ababa and Dire Dawa; and nine regional states, including Tigray, Afar, Amhara, Oromia, Somali, Benishangul-Gumuz, Southern Nations, Nationalities, and Peoples’ (SNNP), Gambella, and Harari. Each regional state has additional divisions known as zones, which are further divided into districts (also known as “woreda") and districts into Kebeles. Figure 1,^1^ shows the spatial plot of Ethiopia’s zones and regions. The regional states are responsible for providing public services because of the transfer of authority to regional governments. The districts oversee service planning and execution, while the regional health bureaus oversee public health administration.

### Source of study data

The dataset utilised in this study included the count of individuals diagnosed with HIV who registered at HIV health care facilities and the number of district-level TB case notifications of individuals who registered in Directly Observed Therapy, Short Course (DOTS). The District Health Office reports the dataset quarterly or yearly to the Federal Ministry of Health (FMoH) via the Health Management Information System (HMIS) [24, 25]. The study dataset, therefore, were obtained from FMoH. In addition to this dataset, the FMoH also provided shape files for district mapping, which were created by Ethiopia’s Central Statistics Agency (CSA).

### Bayesian hierarchical joint spatiotemporal modelling for TB and HIV

In this paper, we assessed the spatiotemporal variation of HIV and TB risks by jointly analysing data of the two diseases obtained from HMIS. We applied a Bayesian hierarchical model approach. The Bayesian hierarchical model estimates the posterior distributions of the parameters in the model by applying a hierarchical order using the Bayes method. In order to estimate the posterior distribution, the observed data via the likelihood function is joined with the prior distributions of model parameters by the Bayes theorem. The Bayesian hierarchical joint spatiotemporal modeling allows the splitting of risks of diseases into two spatiotemporal components: shared and disease-specific.

Let $y_{dit}$ be the *d* disease cases notifications for district *i* in year *t*, $i,\ i = 1, \ldots , M$, $t=1, \ldots ,T$, and $d=1$ and $d=2$ represent HIV and TB diseases, respectively. We assume that each observed case notification $y_{dit}$ follows the Poisson distribution with mean $\mu _{dit}$, and the mean is calculated as $\mu _{dit} = E_{dit} \times \theta _{dit}$ , where $\theta _{dit}$ is the unknown relative risk of *d* disease and $E_{dit}$ is the expected number of *d* disease case notifications. Therefore, $y_{dit} | E_{dit}, \theta _{dit} \sim Poisson(\mu _{dit})$. The expected case notifications $E_{dit}$ represents the number of case notifications for *d* disease that one would anticipate if district *i*’s population behaved like the general population.

Let $N_d$ be the number of general population, then $N_d$ defined as the average of the pooled district population estimates for the study period, i.e., $N_d= \sum _{i=1}^M \sum _{t=1}^T n_{dit} / T$, where $n_{dit}$ is the estimated population of district *i* at time *t* is considered as the population at risk of *d* disease, with $T = 4$ for the current study. The crude rate for *d* disease was then calculated as $\hat{\theta }_{dit} = m_{dit}/n_{dit}$, where $m_{dit}$ is the number of cases notifications of disease *d* in district *i* in year *t*. The expected cases notification $E_{dit}$ for *d* disease was estimated for district *i* in year *t* as $E_{dit} = N_d \times \hat{\theta }_{dit}$ [26]. Then a joint spatiotemporal Poisson regression model for the relative risks $\theta _{dit}$ for the two diseases are defined in a logarithmic scale as

1 $$\begin{aligned} \left\{ \begin{array}{ccc} \eta _{1it} = log(\mu _{1it}) & = & \alpha _1 + \omega _{i} \delta + \phi _{t} \kappa + \nu _{1i} + \gamma _{1t} + \psi _{it}, \\ \eta _{2it} = log(\mu _{2it}) & = & \alpha _2 + \frac{\omega _{i}}{\delta } + \frac{\phi _{t}}{\kappa } + \nu _{2i} + \gamma _{2t} + \psi _{it} \end{array} \right. \end{aligned}$$

where $\alpha _d$ is disease-specific intercepts, $\omega _i$ and $\phi _{t}$ are shared spatial random effect, shared temporal random effect, $\nu _{di}$ and $\gamma _{dt}$ are disease-specific spatial and disease-specific temporal random effects, and $\psi _{it}$ is a common random interaction effect between space and time, $\delta$ is a spatial scaling parameter or weight and $\kappa$ is a temporal scaling parameter or weight. The effects of the shared spatial random effect $\omega _i$ and temporal random effect, $\phi _t$, on the relative risks of HIV and TB are modulated via weights $\delta$ and $\kappa$, respectively [11, 27]. This weighting allows each disease to have its own risk gradients on $\omega _i$ and $\phi _{t}$. Unlike [16], the model in Expression (1) includes a random interaction effect between space and time. The disease-specific spatial and disease-specific temporal effects, $\nu _{di}$ and $\gamma _{dt}$, in the above model allow for departures from any $\omega _i$ and $\phi _{t}$, that is, the diseases HIV and TB may have different spatial pattern or temporal trend. Whereas the common space-time interaction random effect, $\psi _{it}$ provides additional flexibility towards identifying varying patterns.

### Computation and models comparison

Let $\varvec{\nu }_d = (\nu _{d1}, \ldots , \nu _{dN})$ and $\varvec{\gamma }_d = (\gamma _{d1}, \ldots , \gamma _{dT})$ are vectors of disease-specific spatial random effects and disease-specific temporal random effects, respectively, and $\varvec{\omega }_d = (\omega _{d1}, \ldots , \omega _{dN})$ and $\varvec{\phi }_d = (\phi _{d1}, \ldots , \phi _{dT})$ are vectors of shared spatial random effects and shared temporal random effects.

The classical Bayesian inference approach uses the Markov Chain Monte Carlo (MCMC) technique [28]. Since the MCMC technique requires a significant amount of time for analysis, the models in Expression (1) were fitted numerically by applying the integrated nested Laplace approximation (INLA) method [29]. In order to apply a Bayesian method, the stochastic components of the model need prior distributions. We assumed spatially correlated prior distributions for the shared random effects and disease-specific random effects [11]. Specifically, we modeled them using an intrinsic Conditional Autoregressive (iCAR) structure. Using an element of vector $\varvec{\nu }_d$, $\nu _{di}$, iCAR defined as

$$\begin{aligned} \nu _{di} | \varvec{\nu }_{d(-i)}, \tau _{\nu _d}, \textbf{W} \sim Normal \left( \frac{\sum _{j \in \triangle _i} \nu _{dj}}{\mathcal {N}_i}, \frac{\tau _{\nu _d}}{\mathcal {N}_i} \right) , \end{aligned}$$

where vector $\varvec{\nu }_{d(-1)}$ represents the set of disease-specific random effects excluding $\nu _{di}$ for disease *d*, $\tau _{\nu _d}$ is an unknown precision parameter, $\triangle _i$ represents the neighbours of the *i*th district according to the definition of a symmetric binary weights matrix **W**; $\mathcal {N}_i$ is the number of neighbouring districts of the *i*th district and its value also is equal to the sum of the *i*th row of the $\textbf{W}$ matrix [30].

The iCAR model for elements of $\varvec{\omega }_d$ can easily be written following the above definition. In matrix form these can also be defined as

$$\begin{aligned} \varvec{\nu }_d \sim iCAR(\textbf{W}, \tau _{\nu _d} \textbf{I})\qquad \text {and}\qquad \varvec{\omega }_d \sim iCAR(\textbf{W}, \tau _{\omega _d} \textbf{I}),\ d=1,2. \end{aligned}$$

Note that for shared and disease-specific temporal effects, we have used a temporal adjacency structure **Q** [31]. Assume there is yearly fluctuations in elements of $\varvec{\gamma }_d$ and $\varvec{\phi }_d$. Then to reflect this in the priors, in this paper, we have employed a first order random walk (RW1) to model $\varvec{\gamma }_{d}$ and $\varvec{\phi }_{d}$. This modelling also involves the use of a weighted matrix **Q** to define the temporal neighborhood. Or, using $\gamma _{dt} \in \varvec{\gamma }_{d}$, RW1 is defined as

$$\begin{aligned} \gamma _{dt} | \varvec{\gamma }_{d(-t)} \sim \left\{ \begin{array}{cc} Normal(\gamma _{d(t-1)}, \sigma ^2_{\gamma _d}) & \text {for}\ t=1, \\ Normal \left( \frac{\gamma _{d(t-1)} + \gamma _{d(t+1)}}{2}, \frac{\sigma ^2_{\gamma _d}}{2} \right) & \text {for}\ t=2, \ldots , T-1, \\ Normal(\gamma _{d(t-1)}, \sigma ^2_{\gamma _d}) & \text {for}\ t=T; \end{array} \right. \end{aligned}$$

where $\varvec{\gamma }_{d(-t)}$ denotes all elements of $\varvec{\gamma }_d$ except the $\gamma _{dt}$. We used improper flat prior for disease-specific intercepts $\alpha _d$, $d=1,2$. As in [16], $\delta \sim log-Normal(0, 1/5.9)$ and $\kappa \sim log-Normal(0, 1/5.9)$. The space-time random interaction effect, $\psi _{it}$ was specified by a Gaussian exchangeable prior $\psi _{it} \sim Normal(0, 1/ \tau _{\psi }^2)$ where $\tau _{\psi }$ is precision parameter of $\psi _{it}$.

We have considered six priors or hyper-priors for the hyper-parameters of random effects variances $\varvec{\sigma } = (\sigma ^{2}_{\omega },~ \sigma ^{2}_{\phi }, ~ \sigma ^{2}_{\nu _d}, ~ \sigma ^{2}_{\gamma _d}, \sigma ^{2}_{\psi })$ precision parameters $\varvec{\tau } = (\tau _{\omega },~ \tau _{\phi }, ~ \tau _{\nu _d}, ~ \tau _{\gamma _d}, \tau _{\psi })$ used in various literature. Most of these priors are defined using the inverse gamma (IG) distribution [32]. The six priors are

(i) *IG*(1, 0.01), a specification used in [33];
(ii) *IG*(0.001, 0.001), the default prior in the BUGS software [34].
(iii) *IG*(0.5, 0.0005), a specification used in [11];
(iv) *IG*(0.01, 0.01), a specification used in [16]; and
(v) $\Gamma (1, 0.0005)$, the default specification used in inla function of R-INLA package [29];
(vi) A half-Cauchy distribution with scale parameter equal to 25, a specification proposed by [35].

We used sensitivity analysis to determine the most appropriate prior for the study data.

To avoid the identifiability problem in the estimation of intercept, in the computation, we enforced a condition that both the sum of the spatial random effects and the sum of temporal random effects equal to zero [11, 26]. The Bayesian hierarchical joint spatiotemporal analyses were conducted using the function inla() that is available in the R-INLA package [36].

The Deviance Information Criterion (DIC) and conditional predictive ordinates (CPO) were utilised to compare the fitted models. The DIC measures the goodness of fit of a model, and the model with the smallest DIC value is selected as the model that provides the best fit for the data [37]. Whereas the CPO, which is defined as the cross-validated marginal posterior predictive density [38], assesses the predictive capacity of fitted models to the data, where a model with higher CPO value suggests that it has a better predictive performance than other models. However, generally, the CPO value of an observation is close to zero, which was the case in the current study; therefore, the negative sum of cross-validatory predictive log-likelihood [39], i.e., $LS(CPO) = -\sum _{i=1}^M\sum _{t=1}^T \log CPO_{it}$ where $CPO_{it}$ is the CPO of the *i*th district at year *t*, was also used to compare the fitted models for their predictive capacity. Therefore, a model with the lowest *LS*(*CPO*) value has the best predictive performance compared to other models.

### Ethical consideration

The University of South Africa’s School of Science Ethics Committee granted permission for the study (ERC Reference Number: 2021/CSET/SOS/045). Furthermore, the Ethiopian FMoH granted authorization to use their data in the current study. Since we utilised district-level data that have been aggregated, we did not get informed consent from the participants.

## Results

### Exploratory analysis

**Fig. 2:**
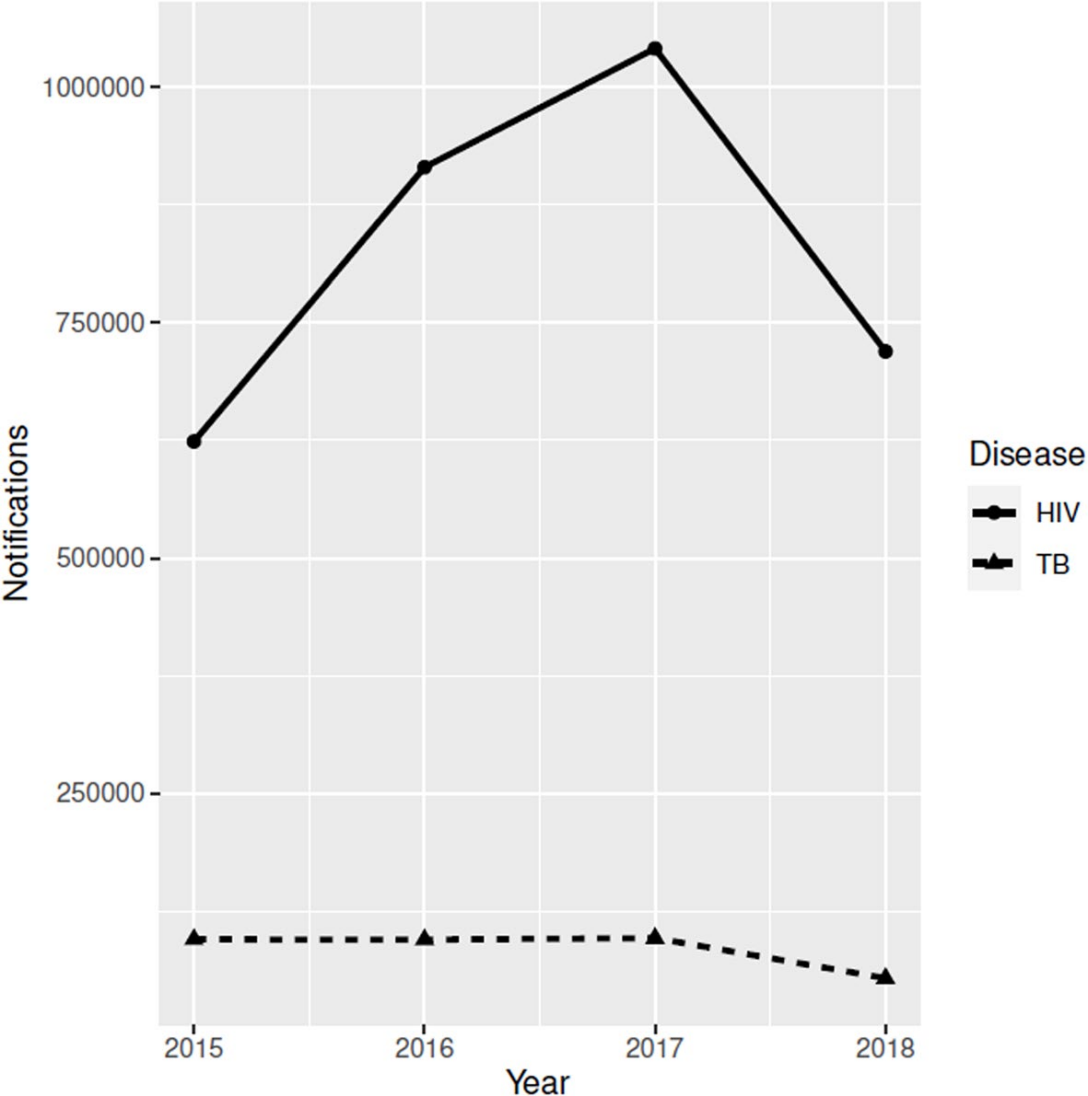
Temporal patterns in number of HIV and TB case notifications

Temporal patterns in the number of case notifications for the two diseases are displayed in Fig. 2. Overall, the TB case notifications in Ethiopia slightly decreased from 94,999 in 2015 to 94,713 in 2016 but increased to 96,300 in 2017 and then decreased to 53,675 in 2018. However, the number of HIV case notifications registered in HIV health care facilities increased from 623,944 in 2015 to 915,015 in 2016 to 1,041,331 in 2017 and decreased to 719,655 in 2018.

**Fig. 3:**
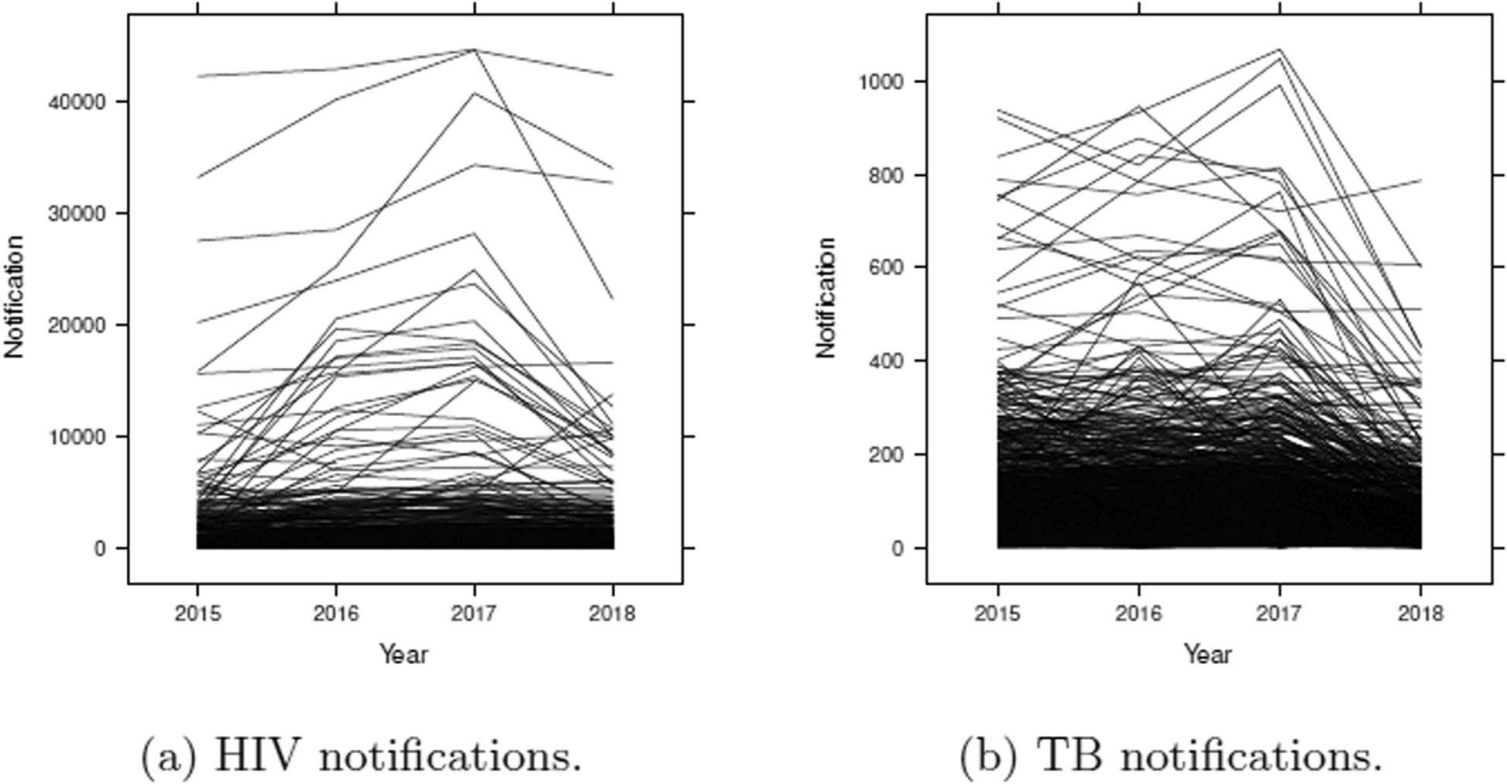
Districts profile plots for number of HIV (panel **a**) and TB (panel **b**) notifications

Figure 3, district profile plots, display the temporal trends of case notifications for each disease in all districts. Meanwhile, most districts have shown a constant increase or stability in the number of reported HIV case notifications during the research period. In addition, there is a non-linear pattern seen for other districts in the country, as shown in (see Fig. 3a). However, the number of TB case notifications for quite a large number of districts had a nonlinear pattern (see Fig. 3b). These suggest varying trends in the number of notifications for HIV and TB by district. Therefore, variations in temporal trends among districts can be attributed to disparities in the underlying causal factors and may vary over time.

The correlations among the district raw standardized incidence rates (SIRs) for the two diseases in 2015, 2016, 2017, and 2018 were 0.415, 0.465, 0.372, and 0.487, respectively. All the correlations are statistically highly significant at a 1% significance level. Since the two diseases are related, our proposal of joint spatiotemporal modeling of HIV and TB or an objective of this study was valid.

**Fig. 4:**
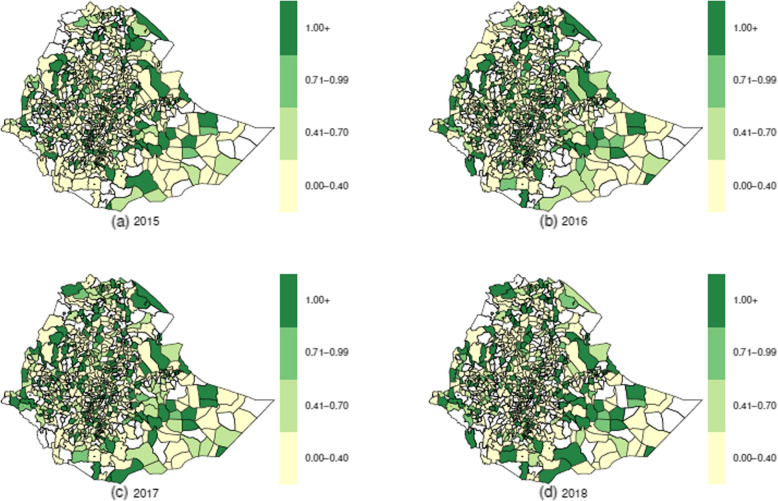
Standardized incidence ratio for HIV

**Fig. 5:**
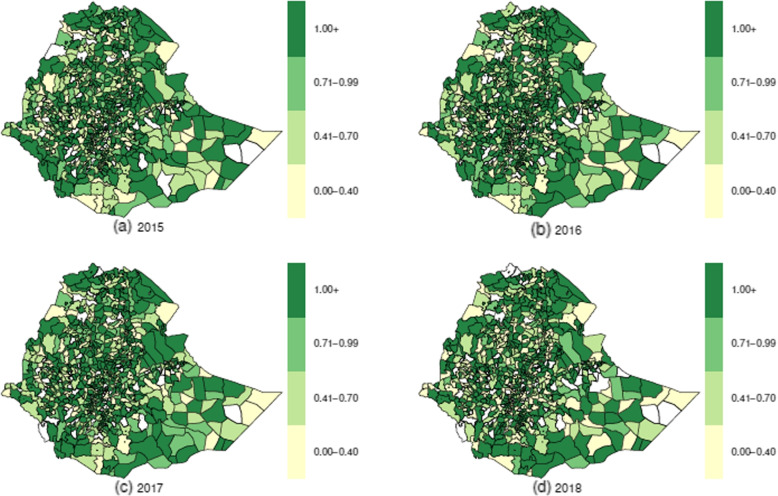
Standardized incidence ratio for TB

The mean annual raw HIV SIRs had inconsistency in the trend; it was 1.454, 1.281, 1.473, and 1.431 in 2015, 2016, 2017, and 2018, respectively. However, the raw TB SIRs had a slightly increasing annual trend; it was 1.229, 1.253, 1.300, and 1.337 in 2015, 2016, 2017, and 2018, respectively. To determine the districts with high risks of HIV and TB, we have produced a sequence of maps for raw (unsmoothed) standardized incidence ratio (computed as Observed / Expected) for each disease, and these are displayed in Fig. 4 for HIV and in Fig. 5 for TB. Although, it was observed that the majority of the districts identified as high-risk for HIV between 2015 and 2018 were also classified as high-risk for TB, maps of HIV (Fig. 4) and TB (Fig. 5) displayed varying spatiotemporal patterns. Both diseases seem to have at least one district of high risk in each region and in each city administration, except Harari region, which had a district of high risk for HIV only in 2018. As seen in Figs. 4 and 5, the progression of the risk during the four years 2015–2018 in TB was significantly faster than that of HIV.

### Sensitivity analysis

The DIC and *LS*(*CPO*) values for each of the six priors of random effects variances precision parameters $\varvec{\tau } = (\tau _{\omega },~ \tau _{\phi }, ~ \tau _{\nu _d}, ~ \tau _{\gamma _d}, \tau _{\psi })$ used for models fitted to the HIV and TB case notifications data are displayed Table 1. Compared to the DIC values of the other models, the *IG*(0.01, 0.01), a specification used in [16], had the smallest value, 47695.36. This model also had the lowest *LS*(*CPO*) value, 6.97637, and the second lowest *LS*(*CPO*), 6.98102, is for the model with the default prior specification used in the inla function of R-INLA package [29], $\Gamma (1, 0.0005)$. Although the difference between these *LS*(*CPO*) values is very small, 0.00465, the difference between their respective DIC values, 7.32, is greater than five, hence, it is a substantial difference [37]. Therefore, the model with the *IG*(0.01, 0.01) prior provided the best fit for the data and had the best predictive performance. Thus, in the sections that follow, we only present findings from model in Expression (1) with *IG*(0.01, 0.01) prior.

**Table 1:** Summary of DIC and *LS*(*CPO*) values of models included in sensitivity analysis

	Type of prior					
	*IG*(1, 0.01)	*IG*(0.001, 0.001)	*IG*(0.5, 0.0005)	*IG*(0.01, 0.01)	*IG*(1, 0.0005)	Half-Cauchy
DIC	47698.69	47698.94	47699.76	47695.36	47702.68	47699.81
*LS*(*CPO*)	6.98187	6.99771	6.99738	6.97637	6.98102	6.98175

The estimated posterior means and the credible intervals (CI) of disease-specific intercepts, derived from the selected prior, are summarised in Table 2. Additionally, the precision parameters of shared and disease-specific spatial and temporal random effects, as well as the interaction random effect of space and time, are presented in Table 2. The table also contains the proportion (percent) variation in the model explained by each random effect. The findings from the chosen model indicate that approximately 53% of the variation in HIV and TB incidences during the study period can be accounted for by the combined influence of the shared temporal component, the disease-specific spatial effect of HIV, and the interaction effect of space and time.

**Table 2:** Summary statistics for estimates of the precision parameters of random effects and coefficients for shared spatial and temporal effects

	Estimates		
Parameter	Mean	95% CI	Percentage of variation
Fixed effect			
$\alpha _1$	−1.529	(−1.591, −1.466)	
$\alpha _2$	−0.134	(−0.153, −0.114)	
Precision parameter			
$\tau _{\omega }$	1.133	(1.046, 1.212)	13.0
$\tau _{\phi }$	1.776	(1.253, 2.631)	20.5
$\tau _{\nu _1}$	1.550	(1.439, 1.749)	17.9
$\tau _{\nu _2}$	0.976	(0.926, 1.017)	11.2
$\tau _{\gamma _1}$	1.200	(1.060, 1.460)	13.8
$\tau _{\gamma _2}$	0.807	(0.680, 1.032)	9.3
$\tau _{\psi }$	1.239	(1.170, 1.505)	14.3
Coefficients for			
Shared Spatial effect			
$\delta _{HIV}$	3.300	(3.042, 3.556)	
$\delta _{TB}$	0.847	(0.736, 0.936)	
Shared Temporal effect			
$\kappa _{HIV}$	0.964	(0.199, 2.656)	
$\kappa _{TB}$	0.582	(0.060, 1.888)	

The posterior means for the coefficients of shared spatial patterns of the two diseases are distinct from one another, and the 95% credible intervals show that the weight of HIV is significantly higher than one. The latter suggests that HIV is more dependent on the shared spatial patterns, hence its spatial pattern closely resembles the shared pattern. The results show that the shared temporal random effect, HIV disease-specific spatial random effect, and the random interaction effect between space and time explain most of the variability in the respective order. The variations explained by HIV disease-specific spatial random effect and temporal random effect are higher than those of TB (Table 2).

### Spatial analysis

**Fig. 6:**
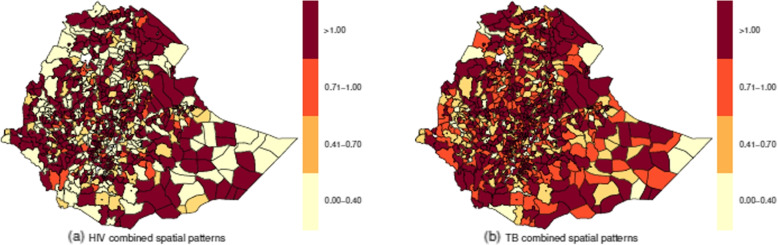
Posterior means of the total spatial effect of HIV (left panel) and TB (right panel)

The relative risk related to the combined spatial patterns for HIV and TB, calculated from the posterior means of total spatial effect, i.e., $\exp (\omega _i + \nu _{di})$, are displayed in Fig. 6. The two diseases show similar spatial patterns in the two city administrations and in some districts, such as at the Western and Eastern Tigray region, Kilbert Raisu (Zone 2) of the Afar region, Central and North Gondar zones in the Amhara region, Asosa zone in the Benishangul-Gomuz region, Anuak zone in the Gambela region, Borena zone in the Oromiya region, Sitti zone in the Somali region including boarder areas in Liben and Shabelle zones in the Somali region.

There were districts identified as high-risk areas within the two city administrations, within all zones of the Afar region; within Awi, east Gojjam, north Gondar, north and south Wollo, and north Shewa zones in the Amhara region; within Asosa and Kemashi zones in the Benishangul-Gumuz region; within Anuak, Mezhenger and Nuer zones in the Gambella region; within the Harari region, Arsi, east Hararge, Horo Guduru Wollega, Illubabor, Jimma, north Shewa and west Shewa zones in the Oromiya region; within Bench Maji, Gamo Gofa, Gurage, Hadiya, Keffa, Sidama and Wolayta zones in the SNNP region; within Nogob and Shebele zones in the Somali region; and central and east Tigray zones in the Tigray region.

The TB high-risk districts most of them were overlap with the HIV high-risk districts, but in some regions, they were different. The districts that are at a high risk of TB were in the two city administrations, in all zones of the Afar region; in east Gojjam, south Wollo, north Gondar and north Shewa zones in the Amhara region; in Kemashi and Metekel zones in the Benishangul-Gumuz region; in Anuak zone in the Gambella region, the Harari region, Arsi, Bale, east Wellega, Finfine zuria, Ilu Aba Bora, Jimma and north Shewa, west Hararge, and west Shewa zones in the Oromiya region; Bench Maji, Gamo Gofa, Gurage, Keffa, Sidama, south Omo and Wolayta zones in the SNNP region; in Afder, Gode and Shinle zones in the Somali region, and central, northwest and south Tigray zones in the Tigray region.

**Fig. 7:**
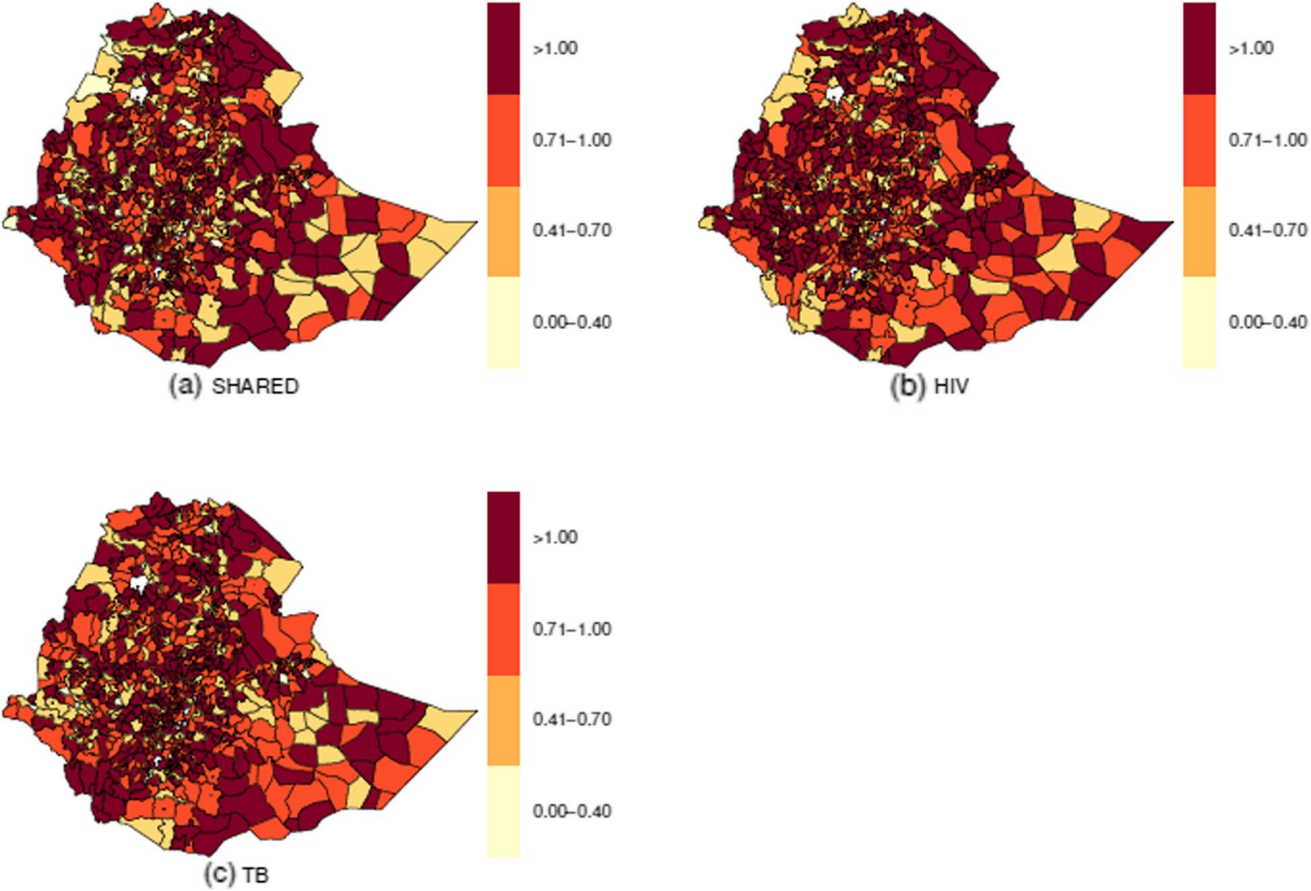
Relative risk of shared spatial patterns ($\omega _{i}$) and disease specific spatial patterns ($\nu _{di}$)

Figure 7 shows the relative risks for the shared spatial effect ($\exp (\omega _i)$) and disease-specific spatial effects ($\exp (\nu _{di})$) patterns. In the three maps, there were common districts of high risk within each region and the two city administrations. On the other hand, when compared to TB disease-specific spatial patterns, the number of districts with a high relative risk ($> 1.0$) of HIV disease-specific spatial patterns is higher, followed by the number of districts with a high relative risk ($> 1.0$) of shared spatial patterns. This may be because HIV is more dependent on the shared spatial term, which means that the shared pattern explains most of the HIV disease-specific patterns. The presence of shared spatial variation or clustering depicted in Fig. 7a can be interpreted as a surrogate for covariates that exhibit spatial variation not included in the model but shared with both diseases [27].

### Temporal analysis

**Fig. 8:**
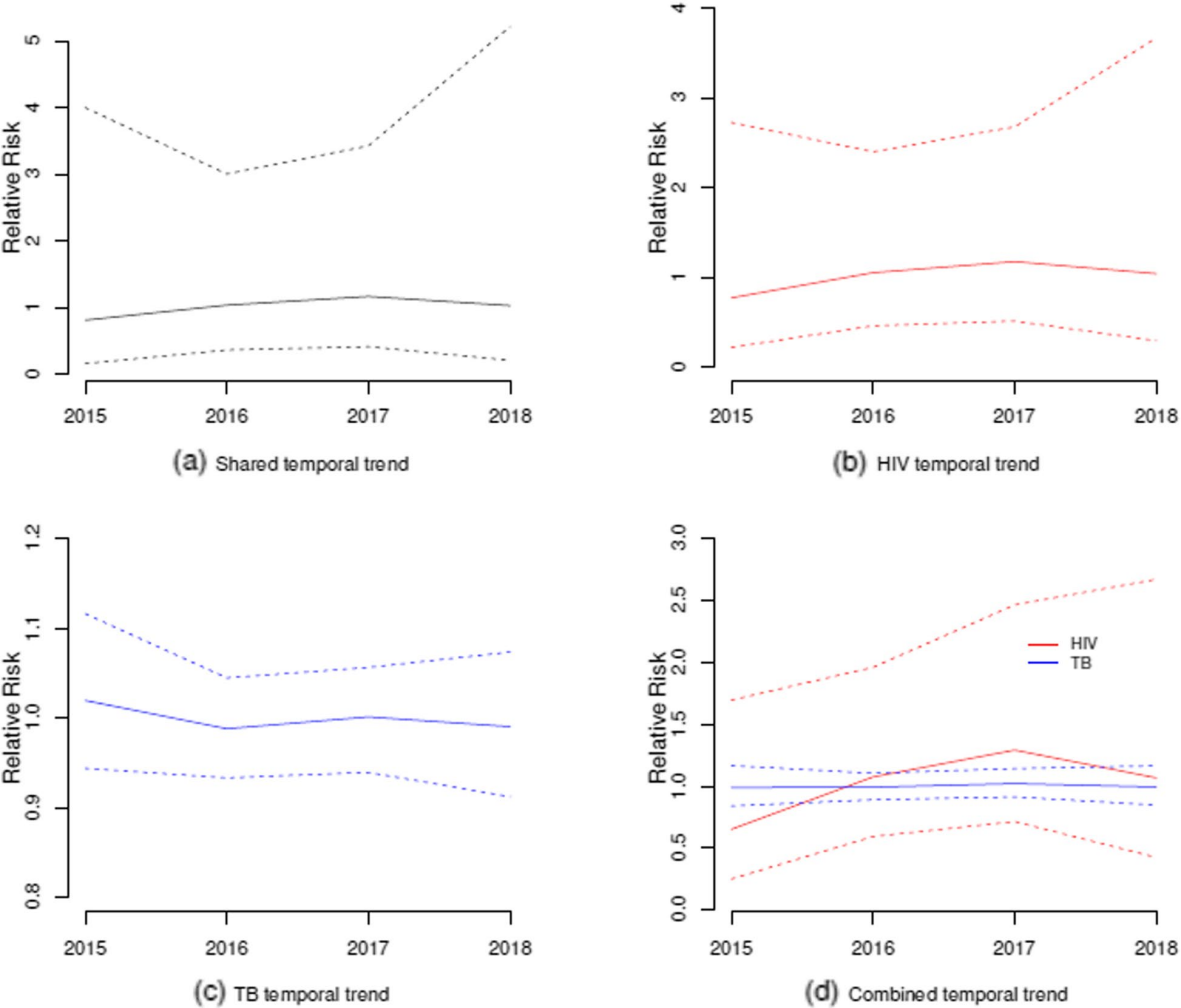
Relative risks of shared temporal effect $\exp (\phi _t)$ (**a**), disease-specific temporal effects $\exp (\gamma _{dt})$ (**b**, **c**) and total temporal effect $\exp (\phi _t + \gamma _{dt})$ (**d**)

The relative risks associated with the shared temporal random effect $\exp (\phi _t)$, disease-specific temporal random effects $\exp (\gamma _{dt})$, and the shared trend effect $\exp (\phi _t + \gamma _{dt})$ are depicted in Fig. 8. The shared temporal random effect suggests a slight increase in risk from 2015 to 2017, then a decrease in risk in 2018. The estimated values of risks are in the interval [0.808, 1.165]. HIV disease-specific temporal effect does not deviate from the shared temporal pattern (its values range between 0.773 and 1.177). However, the specific temporal pattern for TB has a relative risk close to one for the study period (its value ranges between 0.947 and 1.102). The plots in Fig. 8 show that the shared temporal effect almost captures the increasing trend in HIV risk for the first three study years and then a decreasing trend in 2018, whereas the specific effect is negligible because the relative risk estimates are very close to one for all years.

Similar to the shared temporal random effect and HIV disease-specific temporal trend, the combined temporal trend for HIV showed an initial increase in risk over the first three years and then a decreasing trend for 2018 (its values range between 0.653 and 1.293), while the combined temporal trend of TB was almost constant at 1 over the study period (its values range between 0.990 and 1.022). Based on the case notification data, the relative risk of HIV had an increasing trend between 2016 and 2018, and this risk was generally higher compared to the relative risk of TB within the same period.

Table 2 also displays summary statistics of weights $\kappa _d$, $d=1, 2$ for the shared temporal trend. The weight for HIV ($\kappa _1 = 0.964$) was higher than that of TB ($\kappa _2 = 0.582$). Since the weight for HIV is very close to one, this suggests that this disease had a strong dependence on the shared temporal pattern.

### Joint spatiotemporal analysis

**Fig. 9:**
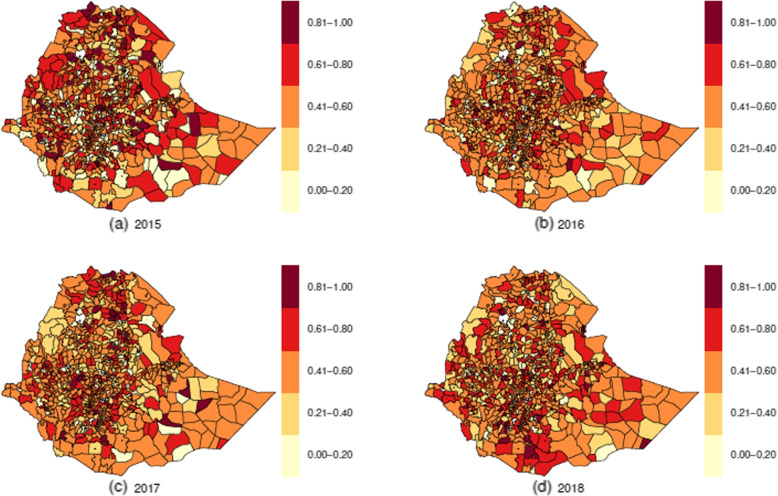
Posterior probabilities of having an estimation of the common space-time interaction term relative risk $\exp (\psi _{it})$ greater than 1, i.e. $P(\exp (\psi _{it})> 1|\textbf{y})$ for each study period

To identify districts with the most substantial space-time interactions, we have produced a map highlighting districts with posterior probabilities of exhibiting a relative risk greater than one (exceedance relative risk) for the smoothed joint spatiotemporal interaction for each study period in Fig. 9. In the figure, if the posterior probability of a district is within the interval [0.81, 1.00], then it is counted as a high-risk area for a disease (HIV or TB) [40]. The number of districts per region with posterior probabilities within the interval [0.81, 1.00] varies between 0 and 21 in 2015 (0 districts in the Benishengul-Gumuz and Harari regions and 21 districts in the Oromia region); between 0 and 14 in 2016 (0 districts in the Afar, Benishengul-Gumuz, Gembella and Harari regions, and 14 districts in the Oromia region); between 0 and 16 in 2017 (0 districts in the two city administrations, Afar, Benishengul-Gumuz, Gambella and Harari regions, and 16 districts in the Oromia region), and between 0 and 11 in 2018 (0 districts in the two city administrations, Benishengul-Gumuz, Gambella, and Harari regions, and 11 districts in the Oromia region). The country had 58, 32, 43, and 27 such districts in 2015, 2016, 2017, and 2018, respectively.

In 2015, relatively more districts with high risks were noticed in east and west Gojjam, south Gondar and south Wollo zones in the Amhara region, Bale, west Hararge, west Shoa and west Wollega zones in the Oromiya region, Gurage zone in SNNP region, east and northwest Tigray zones in the Tigray region. Similarly, in 2016, such districts were observed in north and south Wollo zones in the Amhara region, north and west Shoa, east and west Wollega zones in the Oromiya region; in 2017, north and south Wollo, and north Shewa zones in the Amhara region, east Hararge and west Shoa zones in the Orpmiya region, and central and south Tigray zones in the Tigray region; whereas in 2018, in west Shoa zone in the Oromiya region and Gamo Goffa zone in the SNNP region.

## Discussion

Although WHO-coordinated initiatives have made progress, HIV and TB diseases still have alarming rates of illness and mortality [41]. In the current study, we assessed the joint spatiotemporal variation of HIV and TB risks at the district levels for four years, from 2015 to 2018, in Ethiopia by jointly analyzing data on the two diseases obtained from HMIS. Using a Bayesian hierarchical model, we were able to characterize the spatial and temporal patterns that are shared and disease-specific, as well as the space-time interaction effects of HIV and TB relative risks. It also enabled us to estimate the relative risks of both shared and disease-specific risks, as well as the interaction of space and time effects, and to display these estimates over all the districts in the country.

Results from the exploratory analysis showed that there had been an increase in HIV case notifications registered in HIV healthcare facilities between 2015 and 2017, but there was a sharp decrease from 2017 to 2018; this could be because about 25% of districts failed to submit the HIV case notifications to the national Health Management Information System in 2018. However, TB case notifications had inconsistencies in the annual trend. While the mean raw yearly TB SIRs showed a slightly increasing trend, the raw HIV SIRs showed an inconsistency in the yearly trend. The results also showed that most of the districts identified as high risk for HIV during the study period were also classified as high risk for TB; however, each disease had spatiotemporal variation in the raw relative risk in the four-year study period, 2015–2018. This finding agrees with the FDREMH 2018 report [42]. Except for the Harari region, which had one district of high risk for HIV in 2018, both diseases had at least one district of high risk in each region and in each city administration. There were low reported TB and HIV case notifications in some districts, specifically in the Somali region. Despite the national guidelines recommendation to offer HIV testing to those patients with presumptive TB, there is lag in tracking its implementation in some parts of the country. Therefore, the government may need to implement intensified community-based TB and HIV tests, routine contact investigations, and targeted population-focused interventions.

Overall, the geographic spread of HIV in proportion to TB was more extensive. These findings agree with the results of [43], which indicate that HIV has surpassed TB to a large extent in Mauritania, Senegal, and Gambia. However, the results of the present investigation diverge from those of previous studies in Kenya [26, 44] and [43], which also revealed that TB seemed to be spreading more rapidly than HIV in Rwanda and Burundi. In most districts, the spatial patterns observed in the unsmoothed maps of SIRs for both diseases remained consistent with the spatial patterns of smoothed relative risks. Nevertheless, there were cases where the concentration of high incidence districts became less apparent, for example, for HIV SIR’s districts in the northern part of Fanti Rasu zone (Zone 4) in the Afar region and districts in the south part of Itang zone in the Gambela region in 2015.

In this study, the shared temporal trend showed a marginal increase in risk from 2015 to 2017, followed by a modest decline in 2018. HIV disease-specific temporal pattern had a similar trend to the shared temporal trend. On the other hand, the temporal pattern of tuberculosis risk that was particular to the disease exhibited a level of consistency that was nearly constant throughout the study period. Like the shared and specific temporal trend of HIV, the combined temporal trend for HIV also had a rising risk for the first three years and then a decreasing trend for 2018, while the combined temporal trend of TB was almost constant at 1 over the study period. The case notifications data show that compared to the risk of TB, the temporal trend of the risk of HIV was lower in the year 2015. However, the risk of HIV trend exceeded that of TB over the period 2016 to 2018. These findings agree with the observations made by [44], where in their study, the researchers observed that the rate of HIV risk had a reduced magnitude compared to that of TB during the first two years, but in the last three years, the HIV risk surpassed the TB risk. Although HIV drives TB-related infections, the lower or constant trend of TB in the country during the study period could be due to the government’s effort that all eligible HIV-positive people in areas with a high prevalence of TB should receive TB preventive medication before developing severe manifestations of the virus. Studies in Ethiopia [45, 46] demonstrate this. Other studies in Sub-Saharan Africa also observed similar temporal patterns in TB incidences [47, 48].

There were 174 (22%) districts with similarities in HIV spatial distribution and tuberculosis in the country from 2015 to 2018. Of these districts, 59, 34, 29, 13, and 11 were in the Oromia, SNNP, Amhara, Afar, and Somali regions. The least number of such districts was observed in the Harari region and the Dire Dawa city administration; each had one district. Addis Ababa city administration and the Gambella region had four districts, while the Benishangul-Gumuz and Tigray regions had nine such districts. The large number of districts with high risks of HIV and TB suggests that these districts have inadequate HIV and TB control. If no efficient interventions are developed and implemented, these districts may remain a source of HIV and TB spread. In contrast to the distribution of HIV, which had many districts classified as high-risk areas, the distribution of the shared relative risks was comparable to the distribution of the disease-specific relative risk for tuberculosis. This similarity may be due to a greater dependence on the shared spatial component of HIV, meaning that the HIV spatial patterns are responsible for most of the shared components.

Recall that these two components also had similar temporal trends over the study period. Our results differ from those of [44], whose findings show that there was only a slight difference between the disease-specific risk of HIV and the distribution of the shared risks. However, the distribution of tuberculosis showed a significantly higher number of counties classified as high-risk locations. In addition, other studies in Uganda [44] and China [49] observed the regional clustering of tuberculosis and HIV. However, they applied a co-clustering approach. In the former, the results also show the combined co-clustering of the two diseases. Studies in Brazil reported spatial clustering and temporal trends of HIV incidence [50]. Analyses of spatiotemporal trends of HIV incidence/prevalence in other countries have also demonstrated that the spread of HIV infection varies in both space and time [51, 52].

The posterior probabilities of having an exceedance relative risk for the smoothed joint spatiotemporal interaction were computed for each district and mapped for each study period to identify districts having the most significant spatiotemporal interactions. The results show that interaction components are more prevalent in districts with a relative risk higher than one, suggesting that other factors could play a role in these districts. However, the exceedance risk was consistent around the north and south Wollo zones in the Amhara region, north Shoa, west Shoa, and west Wollega in the Oromia region over most of the study years. Studies conducted in China [49] and Brazil [53] also demonstrated a substantial correlation between the joint risks of both diseases using bivariate maps for the joint distribution of HIV and TB. Furthermore, the findings from the study conducted in Brazil revealed that both diseases are spatially heterogeneous across the country.

Investigating relationships between diseases and geographic space over time is essential to elucidate the extent and severity of the infection and its impact on public health. Such assessment helps to identify priority areas that need control interventions. The effectiveness of infectious disease control efforts is maximized when locations with high rates of reported cases are identified and thoroughly documented. Furthermore, to design highly efficient strategies aimed at decreasing the rates of tuberculosis and HIV transmission, conducting a thorough evaluation of the combined epidemiological patterns of incidences for both diseases, at least at the district level over time. Implementing efficient control measures in regions characterized by a significant likelihood of contracting HIV and TB leads to successful containment of the pandemic [54]. Furthermore, an extensive understanding of high-risk regions is crucial for the effective implementation of surveillance programs and the efficient allocation of resources [55].

Although statistical methods for spatiotemporal analysis of several diseases are well developed, based on the authors’ knowledge, this is the first research on the joint spatiotemporal modeling of HIV and TB incidence notifications data using a Bayesian hierarchical approach in Ethiopia. However, our findings might have been influenced by the following limitations:

1. In the current paper, we used HIV and TB case notification data as a proxy to represent people living with HIV and TB. These data are obtained from subgroups of people who seek medical treatment and care from local healthcare facilities. Therefore, they are indicative of the population residing in the vicinity of these facilities.
2. Since we utilized district-level consolidated data in the study, the conclusions drawn cannot be extrapolated to smaller administrative units in the country, such as Kebele or household levels.
3. It is essential to note that the data on HIV and TB case notifications obtained from the national HMIS may not provide an accurate depiction of the actual prevalence of these diseases in a specific district. This discrepancy could be attributed to cases being underreported or not properly detected.
4. The spatial variations or clustering of the two diseases may be due to different factors not included in the model due to data limitations. However, as stated in the previous section, the shared spatial and temporal components can be interpreted as surrogates for covariates that exhibit spatial and temporal patterns not included in the model but shared with both diseases [27]. At the same time, each disease-specific spatial and temporal component represents those spatially varying and have temporal patterns of risk factors specific to the disease. However, conducting further research that considers additional covariates would be beneficial in exploring the underlying causes of district-level variations. This can be achieved using the joint Bayesian spatiotemporal generalized linear models to examine potential risk factors for TB and HIV infections.

## Conclusion

This paper assessed the joint spatial clustering of HIV and tuberculosis incidence in Ethiopian districts and how they vary over four years, 2015–2018. The Bayesian hierarchical joint spatiotemporal models with shared spatial and temporal random effects, disease-specific spatial and temporal random effects, and interaction between space and time random effect were applied for the analysis. The models allowed borrowing strength across both districts, years, and between diseases to produce smoothed district-level HIV and TB incidence estimates separately for each disease and jointly.

The selected model enabled us to cluster districts that had a high probability of contracting HIV and TB. Overall, the study facilitated the identification of districts with a significant likelihood of contracting HIV and TB, making them a top target for control actions. Moreover, the results of this study could offer Ethiopian health policymakers significant insights for enhancing national, regional, zone, and district strategies in addressing area-specific and comprehensive TB and HIV/AIDS collaborative efforts, as well as in strengthening measures to prevent infection of HIV and TB.

## Supplementary Information

Supplementary Material 1. The R code used for the analysis is available as supplementary material in the “Supplementary Material 1” file.

## Data Availability

The data that support the findings of this study are available from Ethiopian Ministry of Health Office but restrictions apply to the availability of these data, which were used under license for the current study, and so are not publicly available. Data are however available from the authors upon reasonable request and with permission of Ethiopian Ministry of Health Office.

## Footnotes

1 This map was taken from the web site https://en.m.wikipedia.org/wiki/File:Map_of_zones_of_Ethiopia.svg. Note that the new regions South West Ethiopia Peoples’ Region, South Ethiopia Regional State, Central Ethiopia Regional State, and Sidama region are part of the Southern Nations, Nationalities, and Peoples’(SNNP).
